# A pathologic two‐way street: how innate immunity impacts lung fibrosis and fibrosis impacts lung immunity

**DOI:** 10.1002/cti2.1065

**Published:** 2019-06-26

**Authors:** Helen I Warheit‐Niemi, Elissa M Hult, Bethany B Moore

**Affiliations:** ^1^ Department of Microbiology and Immunology University of Michigan Ann Arbor MI USA; ^2^ Department of Molecular and Integrative Physiology University of Michigan Ann Arbor MI USA; ^3^ Department of Internal Medicine Division of Pulmonary and Critical Care Medicine University of Michigan Ann Arbor MI USA

**Keywords:** bacteria, collagen, host defence, macrophage, neutrophil, Toll‐like receptors

## Abstract

Lung fibrosis is characterised by the accumulation of extracellular matrix within the lung and is secondary to both known and unknown aetiologies. This accumulation of scar tissue limits gas exchange causing respiratory insufficiency. The pathogenesis of lung fibrosis is poorly understood, but immunologic‐based treatments have been largely ineffective. Despite this, accumulating evidence suggests that innate immune cells and receptors play important modulatory roles in the initiation and propagation of the disease. Paradoxically, while innate immune signalling may be important for the pathogenesis of fibrosis, there is also evidence to suggest that innate immune function against pathogens may be impaired, leading to dysregulated and/or impaired host defence. This review summarises the evidence for this pathologic two‐way street, highlights new concepts of pathogenesis and recommends future directions for research emphasis.

## Lung architecture

**Figure 1:**
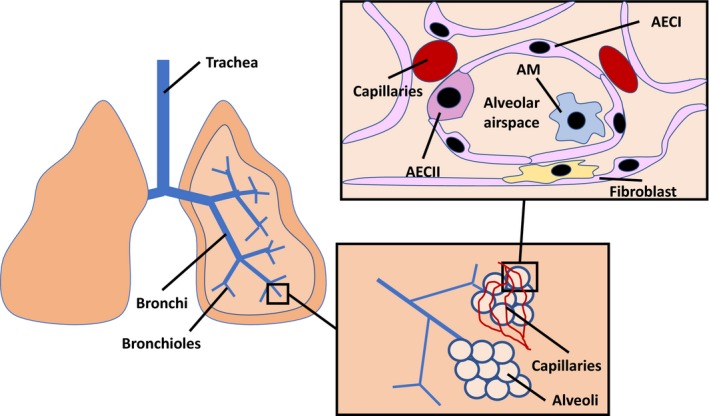
The cellular architecture of the lung. Gas exchange occurs in the alveoli which are surrounded by a network of capillaries. The alveolar airspace is defined predominantly by type I alveolar epithelial cells (AECIs), which are thin and elongated to facilitate the diffusion of oxygen and carbon dioxide. Type II alveolar epithelial cells (AECIIs) function primarily as progenitor cells for AECIs and to secrete surfactant and host defence proteins. Fibroblasts support the structure of the lung and produce extracellular matrix, which contributes to the development of fibrosis. Finally, alveolar macrophages (AMs) reside in the alveolar airspace and are important for maintaining tissue homeostasis and initiating an immune response to respiratory pathogens.

The lung is reportedly made up of dozens of cell types and has evolved architecturally into a series of branching airways and alveoli to support efficient gas exchange (Figure 1).^1^ The ability of the lung to mediate gas exchange requires that the alveoli are characterised by thin layers of type I alveolar epithelial cells (AECs) in close approximation to capillaries to allow efficient cell permeable transfer of oxygen and carbon dioxide.^1^, ^2^ The structure of the lung is supported by a meshwork of fibroblasts, matrix and basement membrane structures.^3^ Type II AECs are important as progenitors for new type I AECs, as a source of surfactant needed to reduce surface tension during respiration and also as a source of host defence proteins.^4^ Finally, the airways are patrolled by innate immune cells including tissue‐resident alveolar macrophages. At homeostasis, there is a fine balance that keeps the airspaces open and cleared of pathogens and debris.^5^ However, following insult or injury to the AECs, exudative inflammation can occur to recruit numerous immune cells. Furthermore, alveolar and endothelial leak postinjury provides a source of profibrotic mediators including growth factors and coagulation components. In most cases, this response is limited and the lung returns to homeostasis, but in other cases, the repair response can become dysregulated leading to lung fibrosis.^6^, ^7^, ^8^

## Lung fibrosis

Lung fibrosis is a condition that is characterised by accumulation of extracellular matrix (ECM) within the lung. When progressive, the accumulating ECM can cause alveolar distortion and loss of gas exchange, eventually leading to respiratory insufficiency. The exact aetiology of most fibrotic lung diseases is unknown (i.e. idiopathic), although there are genetic mutations (e.g. in telomerase or surfactant protein C genes) that can lead to fibrosis and there are environmental insults (e.g. asbestos and silica) that are also known to cause development of lung fibrosis. The most common form of idiopathic interstitial pneumonia is idiopathic pulmonary fibrosis (IPF), and this form of lung fibrosis in humans is the most well‐studied.^9^ IPF has a median survival of 2–3 years, and the disease is more prevalent in older individuals and more common in men than in women. IPF is a chronic and progressive disease believed to be mediated by microinjuries to the lung AECs, chronic inflammation, accumulation of fibroblasts and myofibroblasts, dysregulated wound repair and aberrant deposition of ECM. The clinical course of the disease is highly heterogeneous with some patients showing slow progression and others showing rapid deterioration after events known as acute exacerbations which are believed to be noninfectious.

Despite decades of study, the pathogenesis of pulmonary fibrosis is poorly understood, and there has been ongoing controversy over the role that immune mechanisms may play in disease progression. Most notably, the failure of many immunomodulatory therapies (e.g. augmentation of interferon gamma (IFNγ),^10^ neutralisation of tumor necrosis factor alpha (TNF‐α)^11^ and the early stoppage of the PANTHER clinical trial designed to test prednisone, azathioprine and N‐acetylcysteine due to harm^12^ have led many to conclude that IPF is not caused by immunopathology. However, there has also been an explosion of both human and animal data in recent years to suggest that innate immune cells, in particular, may modify the pathogenesis of lung fibrosis. We will briefly review evidence to support a role for innate immune receptors, inflammasomes, neutrophils and macrophages in mediating development of lung injury and propagation of lung fibrosis. In addition, the fact that immune‐suppression worsened outcomes for IPF patients^12^ highlights the possibility that lung fibrosis may also be modulated, in part, by a weakened immune system. Thus, we will also consider how altered innate immune signalling may lead to recurrent infections and/or alterations in the microbiome which may promote disease. Because lung fibrosis appears to be a final common endpoint for a number of diverse injuries and conditions, we will also explore the impact of innate immunity on two other forms of lung fibrosis, namely silica‐induced toxicity and the development of pneumonitis and fibrosis post‐stem cell transplant and will discuss both preclinical models and data from patients with these conditions.

## Preclinical models to be discussed

Because humans with IPF are generally diagnosed with end‐stage disease, it can be a challenge to study the natural progression of lung fibrosis in humans. Therefore, a number of animal models have been developed that involve chemically induced lung injury, particulates, genetically modified mice, viral infections and radiation. In this review, we will restrict our analyses to three animal models of lung fibrosis, namely intratracheal delivery of bleomycin, silica‐induced lung fibrosis, and a model of viral‐induced pneumonitis and fibrosis following stem cell transplant to highlight the influences of innate immunity on initiation and progression of disease. While these animal models are useful for studies of disease mechanisms that we hope are shared between rodents and humans, it should be acknowledged that none of these animal models fully recapitulate the natural history, time course, histology and pathogenesis of the human diseases.

## Role of innate immune receptors in lung fibrosis

A number of innate immune receptors have been implicated in the pathogenesis of lung fibrosis, and many of these receptors are expressed on both structural and immune cells. One family of receptors is known as pattern or pathogen recognition receptors (PRRs), and they sense ‘danger’ in the form of injury or infection by recognition of pathogen‐associated molecular patterns (PAMPs) or danger‐associated molecular patterns known as DAMPs. This PRR family includes Toll‐like receptors (TLRs), nucleotide‐binding oligomerisation domain receptors (NOD‐like receptors or NLRs), C‐type lectin receptors, retinoic acid‐inducible gene 1(RIG‐1)‐like receptors (RLRs) and cytosolic DNA receptors. PAMP ligands for PRRs include substances such as lipopolysaccharide (LPS), peptidoglycan and flagellin, which are components of bacterial surfaces, but also include unique pathogen genomic structures such as double‐stranded RNA and CpG‐rich DNA that is enriched in microbes. In contrast, the DAMP ligands include substances such as heat‐shock proteins^13^ and fragments of the ECM such as small leucine‐rich proteoglycans, as well as glycosaminoglycans which we recently reviewed.^14^ Interactions of ligands with PRRs ultimately results in signal transduction that changes transcription factors to influence gene expression. For example, many PAMPs induce expression of IL‐6, IL‐1β and CXCL2/8 via induction of nuclear factor kappa‐light‐chain‐enhancer of activated B cells (NF‐kB) to promote inflammatory responses. Similarly, DAMPs derived from ECM can also promote inflammation. For example, fibronectin is a component of provisional matrix and various alternatively spliced forms of fibronectin can trigger TLR4‐dependent NFκB‐mediated release of pro‐inflammatory cytokines from fibroblasts.^15^ Another particularly well‐studied example is the breakdown of high molecular weight hyaluronan (HMW‐HA) following injury into low molecular weight forms (LMW‐HA) that can trigger release of pro‐inflammatory mediators^16^ and also down‐regulate anti‐inflammatory signalling via adenosine A2a receptors.^17^ While these interactions of LMW‐HA with cells like macrophages promote injury and fibrosis, binding to TLR4 on alveolar epithelial cells promotes alveolar repair,^18^ thus helping to restore lung homeostasis. Additionally, production of new HMW‐HA during repair may actively suppress further inflammation by activating T regulatory cells.^19^ When considered together, evidence suggests that signalling via innate immune receptors can play both pathologic and protective roles. While this is likely mediated in part by the kinetics of ligand generation postinjury, there is still much we do not understand about the switch from pro‐injury to pro‐resolution phases.

## The inflammasome in lung fibrosis

The inflammasome is the name given to a multimeric collection of proteins that assemble in response to DAMPs and PAMPs to activate caspase 1 to allow cleavage of pro‐IL‐1β and pro‐IL‐18 into their biologically active forms. There are two main steps involved in activating inflammasomes. The first step is the priming step, and it is mediated by TLR activation to upregulate NF‐kB and induce transcription of inflammasome component genes and also to increase the transcription of pro‐IL‐1β. The second step is the assembly of the multiprotein complex and activation of caspase 1. Release of PAMPs and DAMPs caused by profibrotic injury or infection leads to inflammasome activation, and there are several different kinds of inflammasome scaffolds that can form. Interestingly, it has been suggested that as fibrosis progresses and the lung stiffens, the increased mechanosensing by cells in the lung may lead to ongoing inflammasome activation as a way to perpetuate lung fibrosis.^20^ For example, vimentin has recently been identified as important for inflammasome activation and mice deficient in vimentin are protected from bleomycin‐induced fibrosis and do not stimulate IL‐1β production in response to bleomycin.^21^ This idea of mechanical feed forward innate immune activation is an interesting hypothesis about how fibrosis becomes progressive. Another mystery in the field of IPF research is why the disease is more prevalent in aged individuals. In this regard, it is interesting that the NLRP3 inflammasome is activated in the lung in response to bleomycin^22^ and shows enhanced activation in aged mice relative to young mice.^23^ The NLRP3 inflammasome has also been shown to promote fibroblast activation in response to oxidant injury^22^ and NRLP3^−/−^ mice are protected from bleomycin‐induced fibrosis.^23^ Thus, ageing may predispose mice to worse fibrotic outcomes because they have a lower threshold for inflammasome activation.

In terms of silica‐induced fibrosis, inflammasome activation in macrophages that take up the silica particles is well defined as a critical mediator of the pathogenesis of this disease (e.g. 24, 25, 26). Formation of the NALP3 or NLRP3 inflammasome and pathologic release of IL‐1β is believed to be triggered via the lysosomal damage caused by the uptake of the silica.^25^, ^27^ Similarly, in terms of murine gammaherpesvirus (γHV‐68)‐induced pneumonitis and fibrosis post‐stem cell transplant, the elevated production of prostaglandin E_2_ that is caused by epigenetic alterations post‐bone marrow transplant^28^ has recently been shown to promote prolonged signal 1 for inflammasome activation.^29^ Thus, the enhanced lung injury and prolonged inflammation related to IL‐1β release is likely important in the pathogenesis of all of these models of lung fibrosis.

## Other immune‐related genes and fibrosis

In addition to the pro‐inflammatory signalling by PAMPs and DAMPs described above, genetic studies have suggested that other immune‐modifying genes may also play a role in modulating lung fibrosis, through various mechanisms. For example, the rs5743890 polymorphism in the TOLLIP gene is associated with increased susceptibility to IPF, increased mortality and reduced expression of this protein, which normally functions as a negative regulator of TLR signalling.^30^ Similarly, the gain‐of‐function mutation in the mucin MUC5B gene promoter (rs35705950) leads to overexpression of mucin in the lung and accumulation in terminal bronchioles.^31^ Mucin is believed to play an important host defence function by trapping inhaled particles and pathogens to allow for mucociliary clearance. Some investigators have hypothesised that abnormal accumulation of the protein may overwhelm the mucociliary clearance allowing toxic particles and pathogens to accumulate and be localised next to lung AECs where they may be a source of recurrent injury. In support of this, a mouse genetically engineered to overexpress the mucin, MUC5B, was found to have enhanced bleomycin‐induced lung fibrosis^32^ and these mice display defective mucociliary clearance. Such targeted injury to the AECs may promote fibroproliferation in an attempt at repair. Furthermore, as will be highlighted below, alterations in mucin levels could change the carbon sources within the lung and lead to dysbiosis of the lung microbiota as an additional mechanism for promoting lung fibrosis.

## Innate immune cells and the pathogenesis of lung fibrosis

### Polymorphonuclear leucocytes (PMNs)

In addition to innate immune receptors driving fibrotic pathogenesis, there is also evidence for participation by innate immune cells themselves. Neutrophils or PMNs are short‐lived effector cells that are recruited to sites of injury where they may contribute to lung injury and/or fibroproliferation via the release of proteolytic enzymes such as neutrophil elastase (NE). PMNs are recruited via the release of chemokines, particularly CXCL2/CXCL8, or leukotriene B_4_ (LTB_4_) induced by injury. The presence of increased numbers of PMNs in the bronchoalveolar lavage fluid (BALF) portends a poor prognosis for IPF patients.^33^ Recently, a prospective study also demonstrated that excess PMNs in the blood of IPF patients also predicted worse outcomes and that this enhanced neutrophilia may occur because endothelial colony‐forming cells isolated from IPF patients secreted IL‐8/CXCL8, a known PMN‐recruiting chemokine.^34^ This study also noted PMNs present in histologic analyses of IPF lungs. Excessive PMN degranulation or necrosis can lead to the release of many proteolytic enzymes including NE. It is likely this proteolytic enzyme is critical for promoting fibrogenesis because NE‐deficient mice have reduced levels of fibrosis in response to bleomycin even though these mice showed similar numbers of PMNs recruited to the airspaces as did control mice.^35^ Conversely, histone deacetylase inhibitors that cause significant inhibition of leukotriene A4 hydrolase, a key enzyme needed to generate the PMN‐chemotactic lipid, LTB_4,_ can reduce the initial PMN inflammation seen in response to bleomycin and lessen development of lung fibrosis.^36^

It is also likely that the proteolytic enzymes released by PMNs help to activate other key fibrotic cytokines such as transforming growth factor (TGF)β because bleomycin‐treated NE^−/−^ mice or mice treated with a NE inhibitor, sivelestat, had reduced levels of TGFβ.^35^, ^37^ *In vitro*, NE has been shown to promote fibroblast proliferation and differentiation into myofibroblasts.^38^ Myofibroblasts are a contractile and highly synthetic mesenchymal cell type believed to be a hallmark of fibrotic tissue disease. Another mechanism by which PMNs may promote lung fibrosis is via release of the cytokine IL‐17 during the process of NETosis when PMNs form neutrophil extracellular traps (NETs) that consist of decondensed chromatin, various granules and antimicrobial peptides.^39^ We have previously shown that IL‐17 can directly activate ECM formation and proliferation by lung fibroblasts which express IL‐17 receptors.^40^ It is also noteworthy that in a recent model of acute exacerbation of fibrosis in mice induced by repeated injection of bleomycin at day 21 that both PMNs and IL‐17 were elevated in the BALF.^41^ Taken together, these data suggest that PMNs may contribute to fibrosis via release of NE and NETs and one hypothesised reason that IPF patients may be at risk of increased NETosis to drive fibrogenesis is because they have a deficit of prostaglandin E_2_ (PGE_2_) in the lung.^42^, ^43^ PGE_2_ is a molecule we have previously shown to be an endogenous inhibitor of NETosis.^44^

Silicosis is a fibrotic lung disease caused by toxicity due to inhalation of silica glass particles. PMNs are important for clearance of particulate matter from the lung, and PMNs are capable of phagocytising many different particle types, including silica.^45^ PMNs are also noted to be highly recruited to the lungs of rats exposed to crystalline silica, and this inflammatory response is associated with changes in profibrotic gene signatures.^46^ It is therefore noteworthy that dasatinib, a tyrosine kinase inhibitor that improves outcomes postsilica, is associated with reduced PMN infiltration as well.^47^ Uptake of silica by PMNs is able to cause not only NETosis, but also a form of PMN death called necroptosis, which also results in release of chromatin and cellular contents to facilitate fibrosis.^45^

The data discussed above suggest an important role of PMNs in generating tissue injury and driving fibrogenesis in models of noninfectious acute toxicity caused by bleomycin or silica. In the third model of pneumonitis and lung fibrosis that we are considering in this review, infection of mice at week 5 following a syngeneic stem cell transplant with γHV68 leads to robust chronic inflammation and fibrosis that is highly dependent on IL‐17.^40^ This model is also characterised by infiltration of the lungs by monocyte/macrophages and PMNs. Interestingly, when recruitment of monocytes was reduced by transplanting with CCR2^−/−^ bone marrow, the fibrotic pathology was worse and the number of inflammatory granulocytes, including PMNs was increased, along with higher levels of IL‐17 which as mentioned above can activate fibroblasts.^48^ However, when PMNs were depleted using anti‐GR1 or anti‐Ly6G, the pathologic outcomes were not different.^40^, ^48^ While further work is needed, it seems that in the presence of a replicating pathogen, that innate immune function may be necessary for pathogen clearance^40^, ^48^ and that other granulocytes (e.g. eosinophils) may be able to contribute to tissue damage even in the absence of PMNs. Thinking about potential antifibrotic therapeutics such as anti‐IL‐17, it may be critical that we understand whether or not there is an infectious component to the aetiology of the disease before embarking on treatment.

### Macrophages (tissue resident and inflammatory)

In addition to PMNs, pulmonary macrophages are also thought to play an important role in driving fibrotic pathology and this has been one of the most active areas of recent study. Typically recruited to initial sites of damage or infection by inflammatory signals arising from damage to AECs, pulmonary macrophages are thought to clear pathogens and debris and orchestrate further inflammation by the release of chemokines.^49^ As discussed above, this pro‐inflammatory recruitment of PMNs may result in further lung injury. In normal wound healing responses, however, this pro‐inflammatory phase would be followed by a switch to an immunosuppressive state to help resolve the inflammatory response. This immunosuppressive state is characterised by upregulation of arginase‐1, TGF‐β and IL‐10 to promote repair. If this response is transient, then a return to lung homeostasis is likely. However, if it is prolonged, arginase‐1 metabolism results in the production of proline, a major amino acid component of collagen.^50^ Furthermore, TGF‐β stimulates the production of collagen by fibroblasts.^51^ Our laboratory has recently published data to suggest that IL‐10 can sensitise lung AECs to apoptosis induced by mediators released from activated macrophages.^52^ Thus, prolonged accumulation of these reparative, alternatively activated macrophages may promote fibrogenesis via multiple mechanisms.

While the precise roles of different macrophage subsets in the development of fibrosis have not yet been fully elucidated, studies suggest that certain subsets found within the injured lung may be responsible for driving fibrosis. Monocyte migration appears to be important in fibrogenesis, as pulmonary fibrosis was absent in bleomycin‐treated mice lacking the monocyte chemokine receptor CCR2.^53^ Additionally, circulating Ly6C^hi^ monocytes were shown to enhance the development of fibrosis when adoptively transferred into bleomycin‐treated recipient mice.^54^

More recently, reports have also highlighted the importance of a discrete subset of alveolar macrophages in driving fibrotic lung pathology.^55^, ^56^ In the naïve lung, there exists a self‐renewing population of macrophages that derive from foetal monocytes shortly after birth, referred to as tissue‐resident alveolar macrophages (TR‐AMs), that differ from macrophages that differentiate from circulating bone marrow‐derived monocytes.^57^ Following administration of bleomycin to induce pulmonary fibrosis in mice, a large portion of the alveolar macrophage population (distinguished as Siglec F^+^) is lost.^58^ However, during the fibrotic phase of bleomycin‐induced fibrosis, the numbers of alveolar macrophages increase, concomitant with the appearance of a population of alveolar macrophages expressing lower levels of Siglec F.^58^ Lineage‐tracing systems and transcriptome analyses have characterised these Siglec F^lo^ macrophages as monocytic in origin, supporting the idea that bleomycin‐induced lung injury ablates TR‐AMs which are replaced by monocyte‐derived alveolar macrophages (Mo‐AMs) that migrate into the alveoli and upregulate Siglec F.^55^, ^56^ Expansion of alveolar macrophages has also been observed in human lung tissue from patients with pulmonary fibrosis compared to healthy controls.^56^ Similar to findings in mice, single‐cell RNA sequencing revealed multiple distinct macrophage populations in lungs of patients with fibrosis.^59^

It is likely that the magnitude of TR‐AM ablation dictates the composition of alveolar macrophages remaining following resolution of lung injury. For example, recruitment of Mo‐AMs was observed following diphtheria toxin depletion of CD11c^+^ lung cells,^60^ liposomal clodronate administration^61^ and bleomycin treatment,^56^ but not in mice treated with LPS,^62^ indicating that mass ablation of TR‐AMs after severe lung injury may require differentiation of Mo‐AMs to replenish the alveolar macrophage population. Mo‐AMs localise to sites of fibroblast accumulation following bleomycin‐induced lung injury.^55^ Of note are recent findings identifying direct interactions between macrophages and myofibroblasts via cadherin‐11, which possibly mediate the Mo‐AM localisation to the profibrotic niche,^63^ and importantly, specific deletion of Mo‐AMs was found to ameliorate fibrosis.^55^, ^56^ These findings are further supported in human studies, wherein macrophages isolated from the lungs of IPF patients expressed similar profibrotic genes as their murine Mo‐AM homologs.^55^, ^56^, ^59^

When considering silica‐induced fibrosis, macrophages are the cell type most characterised by inflammasome activation in response to silica^27^ and we discussed above how inflammasome activation may be potentiated by mechanosensing and ageing. In contrast, when examining fibrosis induced by a viral infection post‐transplant, CCR2^−/−^ mice, which fail to recruit circulating monocytes, actually do worse.^48^ These mice also showed higher degrees of viral replication which may exaggerate lung injury to promote fibrosis. Thus, when considering fibrosis complicated by a replicating pathogen, it seems the inflammatory monocytes may serve an important and protective host defence role. Additionally, as mentioned above, if monocyte recruitment is inhibited, as in CCR2^−/−^ mice, granulocyte accumulation is further enhanced with pathologic consequences.

## Microbial drivers of fibrosis

Another way the immune system may impact fibrosis is via regulation of infection and normal microbiota tone. Both empirical clinical evidence and epidemiological studies support a link between IPF and bacterial pneumonia and altered lung microbiota composition. A 2014 study found that IPF patients had double the bacterial burden isolated from BALF compared to healthy controls.^64^ This and more recent reports have shown that baseline bacterial burden predicted the rate of decline in lung function in IPF patients and that higher bacterial burdens correlated with increased risk of death.^64^, ^65^ Another study reported that two common bacterial genera associated with disease progression in IPF are *Staphylococcus* and *Streptococcus*.^66^ *Staphylococcus aureus* and *Streptococcus pneumoniae* are common aetiological agents of bacterial pneumonia, indicating a tangible link between infection and IPF disease progression. The idea of bacterial infection promoting worse outcomes in IPF patients was further supported by the results of a small clinical trial testing the use of the antibiotic co‐trimoxazole. The study found that IPF patients treated with co‐trimoxazole contracted fewer respiratory tract infections and had significantly reduced all‐cause mortality.^67^

**Figure 2:**
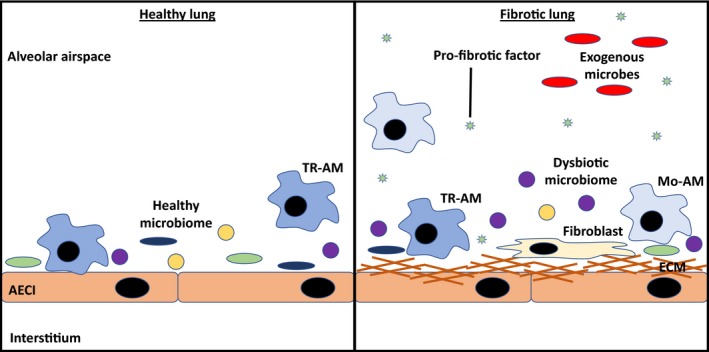
Schematic depicting the changes to the lung innate immune profile and microbiota following the development of fibrosis. In a healthy lung, the alveolar airspace is characterised by tissue‐resident alveolar macrophages (TR‐AMs) and a diverse microbiome with limited soluble mediator production and extracellular matrix (ECM) deposition. In a fibrotic lung, however, monocyte‐derived alveolar macrophages (Mo‐AMs) migrate into the lungs to replace the lost TR‐AMs. Fibroblasts also accumulate and start to lay down ECM which restricts gas exchange and expansion of the lung during inspiration. In addition, there is a dysbiosis of the indigenous microbiome resulting in decreased diversity, enrichment of certain phyla of bacteria, and an overall increase in bacterial burden. These changes are associated with increased levels of profibrotic cytokines and growth factors. Lastly, the presence of certain exogenous microbes has been associated with the development of fibrosis, which could possibly lead to the worsening of disease.

In preclinical work that allows for more mechanistic insight, a striking observation was that germ‐free mice lacking an endogenous microbiome are protected from mortality following bleomycin‐induced fibrosis.^65^ In bleomycin‐treated conventional mice, dysbiosis of the lung community characterised by altered composition and diversity was observed following lung injury and this dysbiosis persisted through the development of fibrosis. Altered microbiota composition in this model was characterised by an increase in the abundance of members of the Firmicute phylum which was sustained all the way through fibrotic development.^65^ In IPF patients, dysbiotic lung bacterial communities correlated with increased levels of profibrotic cytokines and growth factors (IL‐1β, CXCL8, MIP‐1a, GCSF, VEGF, EGF) present in bronchoalveolar lavage fluid.^65^ Taken together, these studies suggest that alterations to the lung microbiome, potentially secondary to dysregulation of innate immune responses, may drive the progression of lung fibrosis. The changes to the lung innate immune profile and microbiota are represented schematically in Figure 2.

We also recently analysed the alterations to the lung and gut microbiome within the murine bone marrow transplantation model of pneumonitis and fibrosis induced by γHV‐68 infection and found that while the process of transplant alone or viral infection alone caused transient dysbiosis, the dual hit was associated with sustained and profound reductions in lung microbial diversity, but there was not a lasting effect on gut microbial diversity.^68^ The major family change seen was a loss of *Lachnospiraceae* in the lungs of mice with pneumonitis and fibrosis. Interestingly, this family is a member of the Firmicute phylum and we have also shown that loss of Firmicutes in the BALF of human hematopoietic stem cell transplant patients that are experiencing pulmonary complications are associated with increased production of numerous pro‐inflammatory cytokines.^68^ This is contrary to what was observed in the bleomycin model of fibrosis,^65^ demonstrating the heterogeneous complexity in mechanisms driving different models of interstitial lung disease. Thus far, the analyses in the bleomycin and bone marrow transplant models are at a high level looking only at phyla and family differences. Future work is needed to determine whether there are particular bacteria that may be protective or pathologic and to understand how the observed dysbiosis may alter production of metabolites such as short‐chain fatty acids which may further modify host cellular responses.

In addition to possible effects caused by changes to the indigenous lung community, there is also an experimental link between fibrotic development and infection with bacterial pathogens. Infection of mice with *Streptococcus pneumoniae* following development of fibrosis was shown to drive greater fibrotic progression than that in uninfected mice.^69^ This was demonstrated in both the adenovirus‐TGF‐β and the surfactant protein C‐diphtheria toxin receptor models of fibrosis, so one hopes that this finding can be extended to more widely used models like bleomycin. In this work, fibrotic progression was found to be mediated by bacterial expression of the cytotoxin pneumolysin, which caused increased apoptotic cell death of AECs and decreased release of prostaglandin E_2._ 69 Interestingly, infection with *Pseudomonas aeruginosa* following bleomycin‐induced fibrosis did not result in fibrotic progression, highlighting the extreme complexity of potential host–pathogen interactions in the fibrotic lung.^70^ Future work is needed to understand whether the ability of bacteria to promote fibrogenesis is mediated solely by a secreted toxin or whether it may involve differential signalling via PRRs and/or differing lung cells targeted by infection.

## Effects of fibrosis on innate immunity

**Figure 3:**
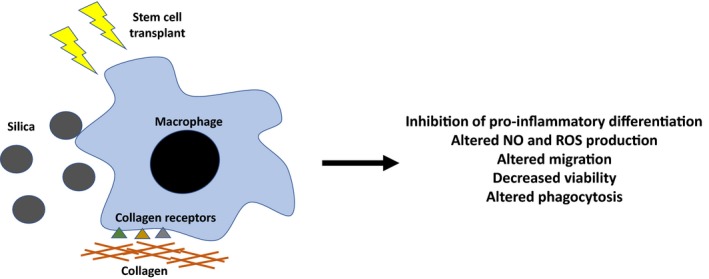
Effects of fibrosis on macrophage function. Evidence suggests that the development of fibrosis may impact the ability of innate immune cells to respond to respiratory infections. Macrophage collagen receptor signalling has been linked to the inhibition of pro‐inflammatory macrophage differentiation and altered migration, nitric oxide (NO) production and phagocytosis. Silica has been shown to decrease macrophage viability and alter phagocytosis, NO and reactive oxygen species (ROS) production. Stem cell transplant impairs macrophage phagocytosis and killing functions due to upregulation of prostaglandin E_2_ production.

Pulmonary macrophages have the ability to drive immune responses to respiratory pathogens by actively phagocytising microbes and producing reactive oxygen intermediates, nitric oxide (NO), TNF‐α and interferons following stimulation.^71^ Macrophages also contribute to immune responses by releasing IL‐1 and IL‐6, and by upregulating MHC molecules to more efficiently present antigen to recruited lymphocytes.^71^ Thus, these responses that can be pathologic for tissue injury are important for host defence. The same holds true for PMNs – many of the responses that drive excessive tissue injury are essential for adequate pathogen clearance. NETs ensnare and kill extracellular microbes,^72^ and NE is an antimicrobial peptide important in defence against Gram‐negative bacteria.^73^ While these responses can be important for initial tissue injury, paradoxically, there is evidence that in established fibrosis, innate immune cells may lose the ability to properly respond to respiratory pathogens (Figure 3).

Collagen is the predominant component of scar tissue and is found in high levels in fibrotic lungs. Macrophages and PMNs have various ways of sensing collagen itself as well as extracellular matrix stiffness, and studies have indicated that the signalling pathways responding to these environmental cues can alter cellular function. Leucocyte‐associated Ig‐like receptor 1 (LAIR‐1), an inhibitory receptor that binds to native collagen, was shown to promote alternative activation of immunoregulatory, tissue‐restorative macrophages and inhibit pro‐inflammatory macrophage differentiation.^74^ LAIR‐1 signalling also reduced human neutrophil NET formation following *ex vivo* treatment with agonistic anti‐LAIR‐1 antibodies.^75^ Mice lacking tyrosine kinase collagen receptors discoidin domain receptors 1 and 2 (DDR1 and DDR2) are known to be protected from pulmonary fibrosis^76^, ^77^; however, these receptors also appear to regulate the function of immune cells. DDR1 signalling is important in macrophage migration and production of NO, two functions key to the ability of macrophages to respond to pathogens.^78^, ^79^ Neutrophil chemotaxis is also regulated by DDR2 when cultured in 3D matrices.^80^ Additionally, transient receptor potential vanilloid 4 or TRPV4, an ion channel sensitive to changes in collagen stiffness, has been shown to regulate macrophage phagocytosis.^81^ Increasing levels of collagen can be found in the lungs as the development of fibrosis progresses, so it is plausible that collagen and matrix stiffness can mediate the function of immune cells through the actions of the aforementioned receptors to alter their responses to respiratory infections.

Similarly, it has been known for more than 30 years that *in vitro* administration of silica to macrophages and neutrophils decreases their viability, likely due to lysosomal disruption, and can limit their ability to phagocytise bacteria and to kill intracellular pathogens.^82^ More recent studies have shown that larger silica particles decrease the ability of alveolar macrophages to produce NO and reactive oxygen species^83^; however, the ultimate impact on cell function relates to the size of the silica particle that is ingested, with submicron particles being more pro‐inflammatory.^84^

With regard to the γHV‐68 model of pneumonitis and fibrosis post‐transplant, the process of transplantation impairs the phagocytic and killing functions of the alveolar macrophages and PMNs^85^ and as discussed above, a failure to recruit inflammatory monocytes impairs clearance of lytic γHV‐68.^48^ However, these alterations may be impacted more by the process of stem cell transplant rather than by the alterations in lung ECM and further research is needed to evaluate this. This model has also been shown to be impacted by alterations in Notch signalling, specifically by loss of delta‐like 4 ligand on dendritic cells and loss of the Notch 1 and Notch 2 receptors on T cells.^86^ This loss of Notch signalling which appears to be a consequence of both the radiation conditioning and the viral infection drives the increased IL‐17 to promote fibrogenesis.

When taken together, there is now a body of literature that clearly supports the important role of innate immune cells in stimulating tissue injury and fibrogenesis, but it also seems that innate immune cells residing in a fibrotic microenvironment may be less equipped to fight off pulmonary infection. We speculate that this two‐way signalling may be another mechanism that promotes the progressive nature of lung fibrosis whereby innate immune cells trigger initial injury, but then undergo reprogramming within the fibrotic niche that impairs host defence, allowing for infections or alterations to the microbiota that ultimately further damage and scar the lung.

## Summary and recommendations for future research

In this review, we summarise the known contributions of the innate immune system to fibrotic progression, highlighting the importance of PMNs and macrophages in driving disease in three unique models of fibrogenesis. The roles of innate immune pattern recognition receptors and inflammasomes were considered. We also discuss the direct contributions of PMNs and macrophages in both lung injury and driving fibroblast proliferation and ECM production to mediate aberrant wound repair. In addition, the role of microbial influences on disease (both commensal and exogenous) is examined. To conclude, we speculate upon how the ability of innate immune cells to respond properly to respiratory pathogens may be compromised following the development of fibrosis.

Despite this compilation of research, much is still unknown about the precise roles of innate immune cells in fibrotic lung disease. While it is thought that fibrosis is perpetuated by injury to the lung, how PMNs fit into the picture is much less clear. Do PMNs drive the initial lung injury or is their presence in the lungs a response to an external stimulus (such as an occult respiratory infection) that initiates the injury? Are they needed for clearance of debris, or do they die in tissue via NETosis and necroptosis to promote fibroblast activation? If so, is this a common mechanism in all forms of tissue fibrosis?

Macrophages are also known to play an important role in regulating wound repair; however, nonpathological wound repair terminates in a return to tissue homeostasis. What is it about the fibrotic microenvironment that dictates whether a macrophage adopts a tissue‐restorative phenotype transiently to allow a return to homeostasis versus becoming pathologically activated to promote fibrosis? In addition, the profibrotic function of the recently characterised Mo‐AM subtype has been suggested,^55^, ^56^ but there is still much to learn about the mechanism of their contributions to disease. While we have discussed some potential hypotheses to explain the progressive nature of IPF and other fibrotic lung diseases, there is no concrete evidence yet for why the disease progresses in humans but is largely self‐resolving in our animal models. Does progressive fibrosis require the presence of human‐residing microbiota or are there important differences in how injury and infection are sensed in mice and humans? While there is a clear association between IPF and the diversity, composition, and overall burden of bacteria found within the lung,^64^, ^65^ we can only speculate on how dysbiosis of the indigenous lung community specifically contributes to disease progression, and whether this dysbiosis is the result of altered immunity or vice versa is still not clear.

Additionally, the complicated interplay between respiratory pathogens and innate immunity in the fibrotic lung is extremely understudied. As one study showed, infection with *Streptococcus pneumoniae* following fibrosis actually increased fibrogenesis through cytotoxin‐mediated AEC death,^69^ supporting a role for bacterial pneumonia driving aberrant wound repair. However, this does not hold true for every bacterial pathogen as *Pseudomonas aeruginosa* did not exacerbate fibrosis,^70^ indicating that this effect is likely pathogen‐specific and highlighting the need for additional research examining host–pathogen interactions in the fibrotic lung. One may speculate that certain bacterial species interact with innate immune receptors to perhaps initiate pro‐inflammatory or profibrotic signalling to promote fibrogenesis, but it must also be remembered that some interactions of DAMPs with TLRs seem to promote repair as well.^18^ How TLR4 signalling may be both restorative and pathologic is not understood.

Lastly, there is a preponderance of evidence to support the idea that the development of fibrosis alters the function of innate immune cells, possibly making IPF patients more susceptible to respiratory infections in the first place. It is well‐known clinically that IPF patients that acquire respiratory infections have very poor outcomes.^12^, ^87^ While respiratory pathogens like *Pseudomonas aeruginosa* may not drive the deposition of additional ECM following infection, they could be the cause of declining lung function in IPF patients due to the induction of inflammation and the inflammatory exudate further limiting gas exchange. This important complication has received relatively little attention in the field of IPF research as there has been a strong focus on studying the ‘noninfectious’ causes of acute exacerbations. Therefore, future effort should consider evaluating the interactions between respiratory pathogens and the innate immune system within the fibrotic lung to better understand how to treat these patients, and to understand that their immune systems may in fact be characterised by impairment, rather than overt activation in late stages of disease.

## Conflict of interest

The authors declare no conflict of interest.
